# Evolution of Stroke Care at Universiti Sains Malaysia Specialist Hospital, Malaysia (1983–2025): Progress, Challenges, and Future Directions

**DOI:** 10.21315/mjms-08-2025-575

**Published:** 2025-10-31

**Authors:** Sanihah Abdul Halim, Mohamad Masykurin Mafauzy, Nur Asma Sapiai, Nasibah Mohamad, Muhammad Ihfaz Ismail, Mohd Hafizuddin Husin, Bazli Md Yusoff, Muhammad Hafiz Hanafi, Muhammad Syahir Mohamad Fauzi, Wan Syahmi Wan Mohamad, Song Yee Ang, Diana Noma Fitzrol, Zaitun Zakaria, Al Hafiz Ibrahim, Wan Mohd Aiman Wan Ab Rahman, Nur Adilah Bokti, Laila Ab Mukmin, Mohamad Hasyizan Hassan, W Mohd Nazaruddin W Hassan, Izzat Ismail, Rose Izura Abdul Hamid, Saidatul Manera Mohd Daud, Zulzamri Ismail, Salmi Zura Mohd Salleh, Rosni Mane, Kamarul Aryffin Baharuddin, Mohd Shafie Abdullah, Ab Rahman Izaini Ghani, Zamzuri Idris, Jafri Malin Abdullah

**Affiliations:** 1 Department of Internal Medicine (Neurology), School of Medical Sciences, Health Campus, Universiti Sains Malaysia, Kubang Kerian, Kelantan, Malaysia; 2 Department of Emergency Medicine, School of Medical Sciences, Health Campus, Universiti Sains Malaysia, Kubang Kerian, Kelantan, Malaysia; 3 Department of Radiology, School of Medical Sciences, Health Campus, Universiti Sains Malaysia, Kubang Kerian, Kelantan, Malaysia; 4 Department of Neurosciences, School of Medical Sciences, Health Campus, Universiti Sains Malaysia, Kubang Kerian, Kelantan, Malaysia; 5 Rehabilitation Medicine Unit, School of Medical Sciences, Health Campus, Universiti Sains Malaysia, Kubang Kerian, Kelantan, Malaysia; 6 Department of Anaesthesiology and Intensive Care, School of Medical Sciences, Health Campus, Universiti Sains Malaysia, Kubang Kerian, Kelantan, Malaysia; 7 Universiti Sains Malaysia Specialist Hospital, Kubang Kerian, Kelantan, Malaysia; 8 Brain and Behaviour Cluster, Universiti Sains Malaysia Specialist Hospital and School of Medical Sciences, Health Campus, Universiti Sains Malaysia, Kubang Kerian, Kelantan, Malaysia; 9 Neurology Unit, Department of Medicine, Hospital Raja Perempuan Zainab II, Kota Bharu, Kelantan, Malaysia

**Keywords:** Universiti Sains Malaysia, comprehensive stroke centre, stroke care, history and evolution

## Abstract

Stroke remains a significant global health burden and is the third leading cause of mortality in Malaysia. Despite advances in the management of acute ischaemic stroke through reperfusion therapies, such as intravenous thrombolysis and endovascular thrombectomy, access to these treatments remains limited in many parts of Malaysia due to high costs and infrastructural constraints. Universiti Sains Malaysia (USM) Specialist Hospital, located in Kelantan state, has transitioned into a comprehensive stroke centre, particularly addressing the needs of suburban and rural populations. USM Specialist Hospital has implemented a multidisciplinary, protocol-driven stroke service that encompasses a rapid triage system, 24-h neuroimaging, reperfusion therapies, a dedicated acute stroke unit, neurocritical care services, cerebrovascular neurosurgery services, and a comprehensive rehabilitation programme. This coordinated stroke management model has improved clinical outcomes, patient survival, and functional recovery rates. The USM Specialist Hospital experience highlights the importance of structured, multidisciplinary stroke care pathways in enhancing treatment access and optimising outcomes, particularly in resource-limited healthcare settings.

## Introduction

Stroke, a major global health challenge, encompasses two primary categories: ischaemic and haemorrhagic types. Ischaemic stroke, which accounts for approximately 85% of all stroke cases, is currently the third leading cause of mortality in Malaysia, following ischaemic heart disease and pneumonia (1). Despite the lower incidence of haemorrhagic stroke, it typically has a more severe course and is associated with higher morbidity and mortality rates. National data revealed that the overall stroke-related mortality rate is 11.1%, with a three-fold increase for haemorrhagic stroke compared with that for ischaemic stroke (2). Globally, the advent of reperfusion therapies, including intravenous thrombolysis and endovascular thrombectomy (EVT), has significantly improved outcomes in ischaemic stroke (3). However, in Malaysia, the high cost and limited accessibility of these treatments remain the primary challenge.

Universiti Sains Malaysia (USM) Specialist Hospital, a tertiary care and teaching hospital, is located in the state of Kelantan on the east coast of Peninsular Malaysia. It primarily caters to a predominantly suburban and rural population. In 2024, it recorded a total of 1,047 acute ischaemic stroke admissions. Raja Perempuan Zainab II Hospital, another major tertiary care centre in Kelantan, managed 1,153 cases. An additional 727 patients were treated across six district hospitals within the state. A local study conducted at USM Specialist Hospital among patients with acute stroke, before the introduction of reperfusion therapies, revealed survival rates of 78% at 28 days, 74% at 1 year, and 71% at 5 years after a stroke event (4).

## Recent Development of Stroke Services at USM Specialist Hospital

Since its establishment in 1983, USM Specialist Hospital (formerly known as Hospital USM until its rebranding in 2025) has evolved into a leading comprehensive stroke centre on the east coast of Peninsular Malaysia. It is the only facility in the region offering advanced reperfusion therapies for acute ischaemic stroke, including intravenous thrombolysis and endovascular thrombectomy. The centre adopts a multidisciplinary team approach, integrating the expertise of emergency physicians, neurologists, interventional radiologists, neuroradiologists, neurosurgeons, neuro-intensivists, and neurorehabilitation specialists. A standardised stroke protocol, coupled with a rapid triage system, facilitates the timely identification and early intervention for acute stroke cases.

This coordinated stroke care pathway is supported by advanced diagnostic capabilities, including 24-hour on-site computed tomography (CT), perfusion imaging, and magnetic resonance imaging (MRI) (in selected cases), as well as a specialised acute stroke unit and a high-dependency unit for focused medical management, and a neuro-intensive care unit for critically ill patients, complemented by comprehensive post-stroke rehabilitation services.

The centre is supported by a robust cerebrovascular neurosurgical service, which plays a pivotal role in managing complex stroke cases. This includes revascularisation procedures for acute large vessel occlusions, surgical intervention for haemorrhagic strokes, decompressive craniectomy for malignant cerebral infarctions, and the management of cerebrovascular malformations. The implementation of this integrated stroke care model has led to measurable improvements in clinical outcomes, including reduced door-to-needle and door-to-groin times for reperfusion therapies, as well as improved survival rates and functional outcomes among patients with acute ischaemic stroke.

## History and Evolution of Stroke Services at USM Specialist Hospital (1983–2025)

The initial stroke services laid the foundation for the current advancements in stroke care. The development of stroke services can be delineated into several distinct phases.

### Establishment and Foundation (1983–1994)

In its early years, stroke care at USM Specialist Hospital was limited to acute medical management provided within the general wards. Services were supported by local general physicians, visiting neurologists, and neurosurgeons from abroad (5). At that time, stroke care was limited to bedside clinical diagnosis, basic neuroimaging, and standard medical and surgical treatments, alongside clinical teaching for both undergraduate and postgraduate students. Despite limitations, these early efforts laid the groundwork for the development of future structured stroke care pathways.

Among the neurologists and neurosurgeons who contributed during this era were Dr. Chandrasekar Bhallachandra Khare (India, 1983–1988) and Dr. Pratap Chand (India, 1983–1988), Dr. Fauzi Ahmad Ali Salem (Egypt, 1984–1987), Dr. Benedict Marius Selladurai (Sri Lanka, 1990–1993), Dr. Shanmugan Chandrasekaran (India, 1992–1996), Dr. Maung Nyunt Win (Myanmar, 1990–2000) and Dr. Hanifah Abdul Ghafoor (1993–1995) (5, 6).

### Expansion and Consolidation (1995–2005)

**Figure 1:**
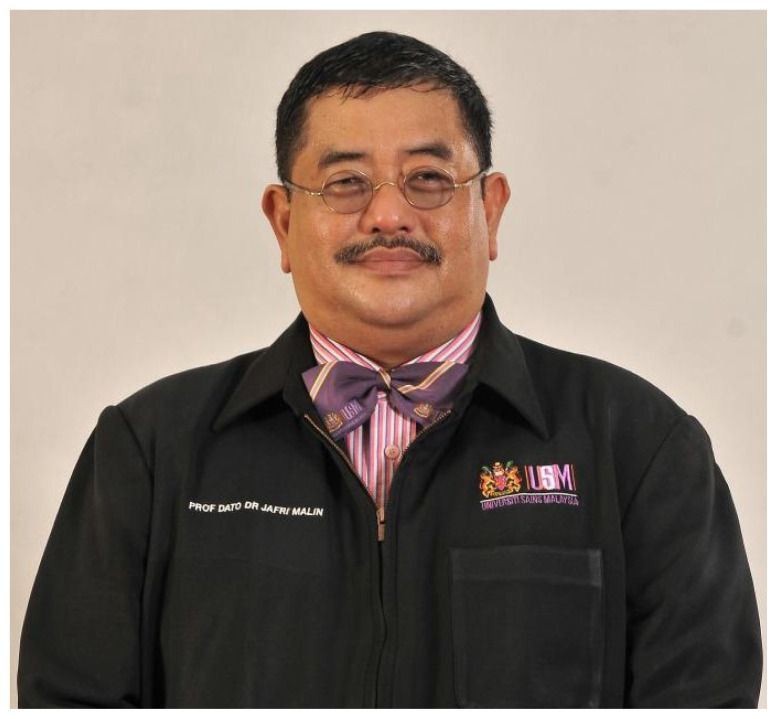
Professor Dato’ Dr. Jafri Malin Abdullah is the founding pioneer of the Department of Neurosciences and the subspecialty training programme in neurosurgery at the School of Medical Sciences, Universiti Sains Malaysia

**Figure 2:**
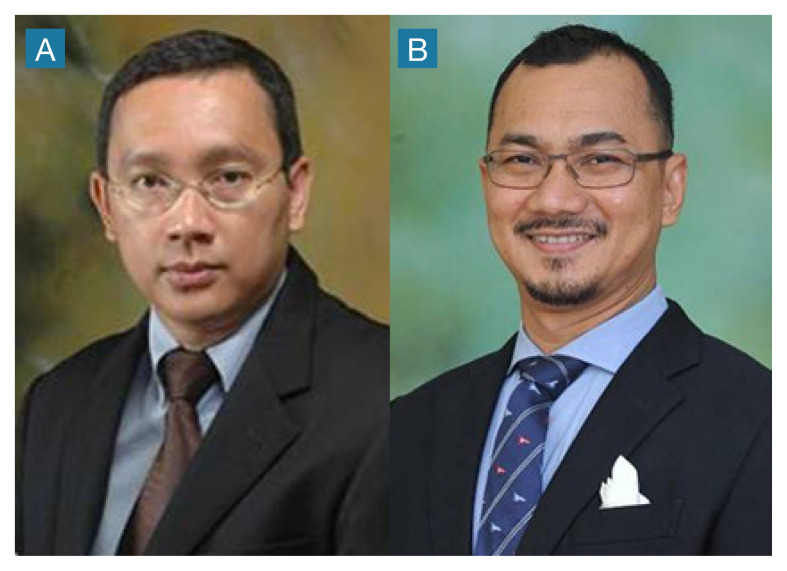
Current senior consultant neurosurgeons at USM Specialist Hospital: A) Professor Dr. Zamzuri Idris and B) Professor Dato’ Dr. Ab Rahman Izaini Ghani

This era marked a significant period of expansion in neuroscience and stroke services at USM Specialist Hospital. In 1995, Professor Dato’ Dr. Jafri Malin Abdullah (Figure 1) became the first local neurosurgeon to serve at the hospital (6). Subsequently, the Department of Neurosciences was established in 2000, serving as a dedicated centre for postgraduate neurosurgical training and a research hub for both basic and clinical neurosciences (6). The department also facilitated in-house training to increase the number of locally trained neurosurgeons at USM Specialist Hospital (Figure 2). Among them were Professor Dr. Zamzuri Idris (training period: 2001–2005) and Professor Dato’ Dr. Ab Rahman Izaini Ghani (training period: 2003–2006) (6). Other locally trained neurosurgeons who have served at the hospital include Dr. Sani Sayuthi (2001–2009), Professor Dato’ Dr. Mohamed Saufi Awang (2001–2010), and Professor Dr. Badrisyah Idris (2004–2019).

During this period, neurosurgical stroke services underwent substantial advancement, particularly in the management of haemorrhagic stroke and intracranial aneurysms. The establishment of a dedicated neurocritical care unit equipped with advanced physiological monitoring systems enabled the implementation of multimodal therapeutic approaches for managing acute neurocritical conditions (7, 8). Neurosurgeons managed hypertensive intracerebral haemorrhages through craniotomy and hematoma evacuation and treated spontaneous intraventricular haemorrhages using intraventricular streptokinase administration or endoscopic lavage. Additionally, decompressive craniectomy with expansile duraplasty was performed for malignant middle cerebral artery (MCA) infarctions (9, 10).

**Figure 3:**
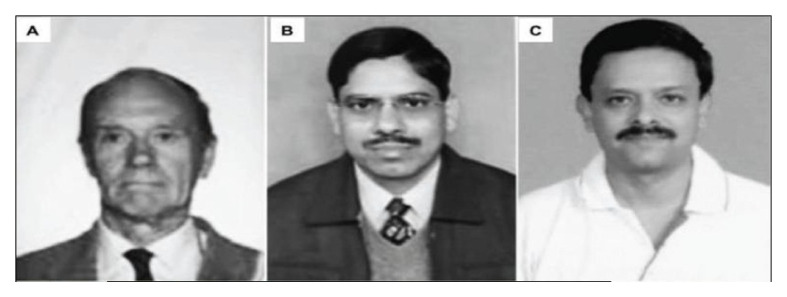
Former visiting neurosurgeons: A) Professor Luc Calliauw (2004), B) Dr. Raj Kumar (2005), and C) Dr. Hillol Kanti Pal (2005)

Among the neurologists who contributed during this era were Dr. Mohd Riduan Abdullah (1994–1998), Dr. Zakaria Abd Kadir (1995–1999), and Dr. Anup Kumar Thaker (India, 1997–1998). In 2002, two neurologists from India, Dr. John Tharakan and Dr. Atul Prasad, joined and expanded the neurology and stroke services. The neurosurgeons (Figure 3) include Dr. George Jain Panattil (India, 2002–2005), Dr. Prakash Rao Gollapudi (India, 2004–2005), Professor Luc Calliauw (2004), Dr. Raj Kumar, and Dr. Hillol Kanti Pal (2005) (5, 6).

In addition, the introduction of neurointerventional radiology services by Professor Dr. Mohd Shafie Abdullah in 2003, following his return from subspecialty training, significantly enhanced the cerebrovascular therapeutic capabilities at the USM Specialist Hospital. This advancement facilitated the adoption of minimally invasive endovascular procedures for treating intracranial aneurysms and other cerebrovascular anomalies, thereby expanding the institution’s capacity to manage complex neurovascular disorders with reduced procedural risk and improved patient outcomes.

### Training and Specialisation (2006–2016)

**Figure 4:**
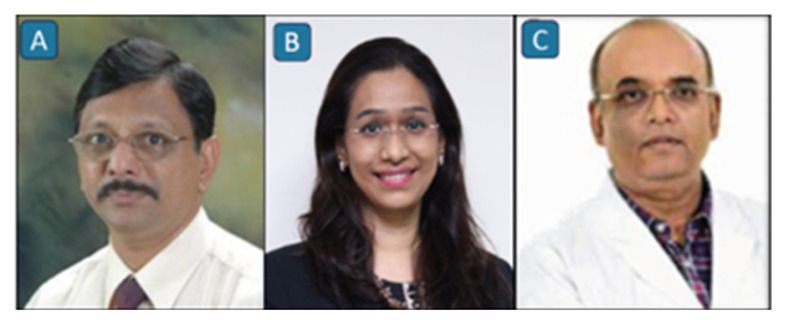
Former Consultant Neurologists at USM Specialist Hospital: A) Professor Dr. John Tharakan, B) Associate Professor Dr. Shalini Bhaskar, and C) Associate Professor Dr. Atul Prasad

The Department of Neurosciences, in collaboration with the School of Medical Sciences, actively supports the expansion of subspecialty training to strengthen academic capacity and increase the number of qualified trainers within USM. Under the leadership of Professor Dr John Tharakan (Figure 4A), the department successfully launched the Advanced Master of Medicine (Neurology) Programme, marking a significant milestone in the development of neurology subspecialisation at USM (11). Associate Professor Dr. Shalini Bhaskar (Figure 4B), a graduate of this programme, completed her subspecialty training from 2007 to 2010, and subsequently contributed to the growth of neurology and stroke services at the institution (11).

Professor Tharakan and Dr. Shalini jointly developed USM’s first institutional stroke thrombolysis protocol in 2007. Despite this important milestone, awareness and acceptance of acute stroke thrombolysis among healthcare providers and the public remained limited during the early phase of implementation. Between 2012 and 2016, the average number of patients receiving intravenous thrombolysis was only 2.5 cases per year.

To address the critical shortage of local neurologists, the School of Medical Sciences granted Dr. Sanihah Abdul Halim to undergo subspecialty training in neurology from 2013 to 2015. However, Dr. Shalini left USM in 2015, and following the retirement of Professor Tharakan in 2017, Dr. Sanihah became the sole neurologist at the institution. She continued to lead the advancement of clinical neurology and acute ischaemic stroke care at USM.

Concurrently, neurosurgical services at USM experienced significant growth. The Department of Neurosciences gained international recognition when it was accredited by the World Federation of Neurosurgical Societies as the 17th Advanced Postgraduate Training Centre globally, with a specific emphasis on stroke surgery training (6). A major achievement occurred on 19 June 2014, when the department successfully performed its first microsurgical clipping of an anterior communicating artery aneurysm using indocyanine green video-angiography, facilitated by a Zeiss high-resolution surgical microscope. This advancement, driven by the Advanced Neuroscience Initiative under the leadership of then-Vice-Chancellor Professor Dato’ Omar Osman, marked a transformative step in the development of cerebrovascular neurosurgical capabilities at USM.

### Integration of Advanced Stroke Care Towards a Comprehensive Stroke Centre (2017–2025)

During this period, ischaemic stroke care underwent substantial transformation, driven by the integration of multidisciplinary services and the implementation of evidence-based clinical practices aligned with international stroke guidelines (12). A key component of this advancement was the establishment of a dedicated multidisciplinary stroke team, which enabled coordinated management across the entire continuum of acute stroke care.

#### Stroke Team at USM Specialist Hospital

**Figure 5:**
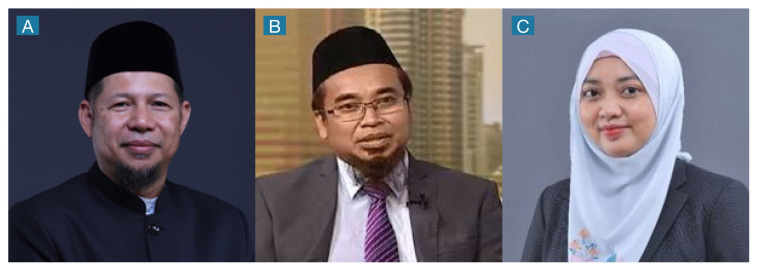
Pioneers of the Multidisciplinary Stroke Team: A) Professor Dr. Kamarul Aryffin Baharuddin, Consultant Emergency Physician; B) Professor Dr. Mohd Shafie Abdullah, Consultant Interventional Radiologist; and C) Associate Professor Dr. Sanihah Abdul Halim, Consultant Neurologist

In 2017, the stroke team at USM Specialist Hospital was established with the primary objective of enhancing knowledge and implementing the reperfusion therapies in acute ischaemic stroke. The initial multidisciplinary team (Figure 5) comprised an emergency physician (Professor Dr. Kamarul Aryffin Baharuddin), an interventional radiologist (Professor Dr. Mohd Shafie Abdullah), and a neurologist (Associate Professor Dr. Sanihah Abdul Halim). Over time, the team expanded to include rehabilitation physicians and therapists, radiologists, neurosurgeons, neuro-intensivists, and dietitians, fostering a comprehensive and collaborative approach to stroke care (13).

During this formative period, the team identified that a major barrier to effective stroke management was the limited awareness and understanding of stroke among healthcare providers and the public (14, 15). In response, the stroke team launched a series of targeted stroke awareness campaigns to improve stroke recognition, increase familiarity with evidence-based reperfusion strategies, and promote primary and secondary stroke prevention.

**Figure 6:**
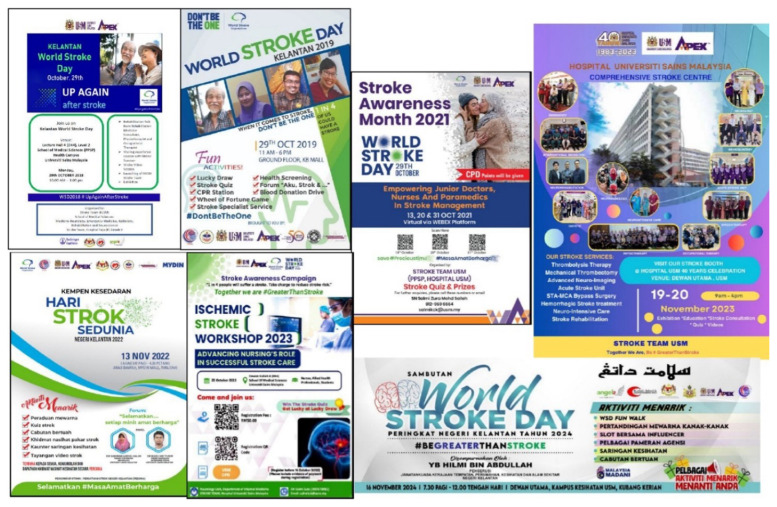
Stroke awareness programmes organised by the Stroke Team USM from 2018 to 2024, highlighting various community engagement initiatives, educational campaigns, and public health activities

**Figure 7:**
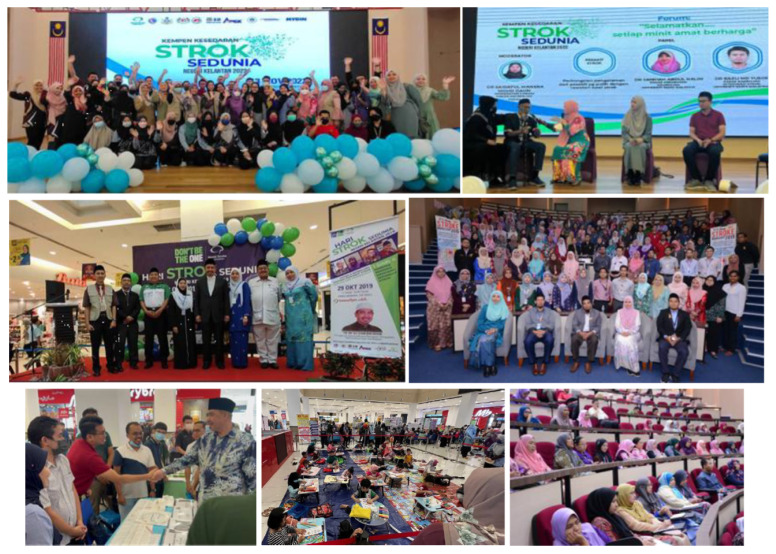
Stroke awareness activities conducted during various awareness programmes, including seminars, forums, exhibitions, and colouring contests for children, aimed at educating the public and promoting stroke prevention across all age groups

Annual stroke awareness programmes (Figures 6 and 7) were organised in alignment with the global themes set by the World Stroke Organization, aiming to improve stroke awareness among healthcare professionals and the public. The first institutional awareness campaign, *Kelantan World Stroke Day 2018*, was conducted by the USM stroke team under the theme #UpAgainAfterStroke. This educational event, held in Lecture Hall 4 at USM, primarily targeted healthcare providers.

In 2019, two major events were held: a stroke workshop for healthcare professionals at USM and a public awareness campaign, *Kelantan World Stroke Day 2019*, under the theme “When It Comes to Stroke, #Don’tBeTheOne.” The latter took place at KB Mall, Kota Bharu, and was officiated by the Kelantan State Health Executive Councillor, YB Dato’ Dr. Izani Husin.

Due to the COVID-19 pandemic, the 2021 Stroke Awareness Month Programme was conducted virtually under the theme #SavePreciousTime. It featured a series of three weekly webinars comprising expert lectures and interactive forums to maintain engagement despite physical restrictions.

In 2022, another World Stroke Day campaign was organised at Mydin Mall, Kota Bharu, in collaboration with the Kelantan Stroke Society (a non-governmental organisation). The event was officiated by the State Health Executive Councillor and focused on increasing public awareness and promoting stroke prevention.

In 2023, the stroke team conducted two key initiatives: a one-day ischaemic stroke nursing workshop to enhance nursing competencies in stroke care, and a public stroke awareness exhibition held during the Hospital USM 40th Anniversary Celebration under the theme #BeGreaterThanStroke. The exhibition highlighted early stroke recognition, prevention strategies, and recovery support (16).

In 2024, the team collaborated with the Kelantan State Health Department and local NGOs to organise World Stroke Day activities, including a fun walk, educational exhibitions, and community based health education programmes.

Additionally, the stroke team also partnered with the Ministry of Health (MOH) hospitals in Kelantan and the Kelantan State Health Department to strengthen regional stroke care delivery. In 2022–2023, the team secured an industry grant from Penumbra Inc. in collaboration with the Division of Industry and Community Network and the School of Medical Sciences, USM.

**Figure 8:**
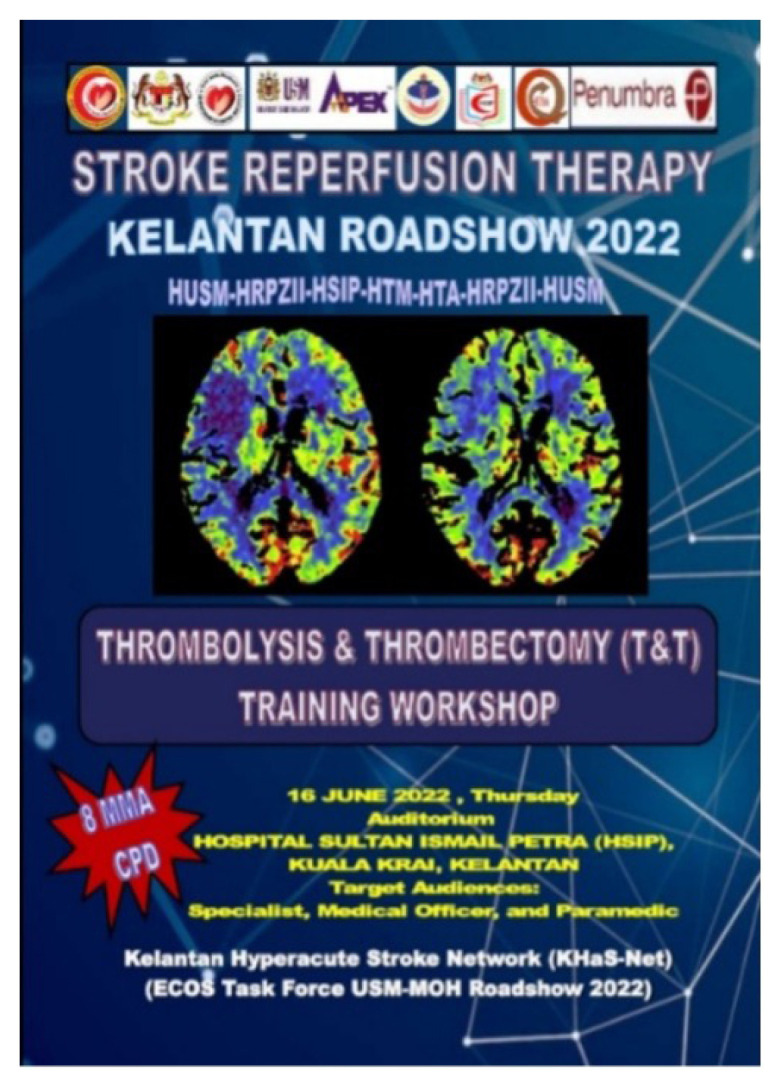
Stroke Reperfusion Therapy Kelantan Roadshow (2022–2023), a statewide initiative focused on training healthcare professionals in major hospitals on the administration of thrombolysis and the role of thrombectomy in the management of acute ischaemic stroke

Led by Associate Professor Dr. Sanihah Abdul Halim, the grant supported the “Stroke Reperfusion Therapy Kelantan Roadshow 2022–2023” initiative (Figure 8), aimed at promoting the implementation of reperfusion therapies, both intravenous thrombolysis and endovascular thrombectomy, across the state.

**Figure 9:**
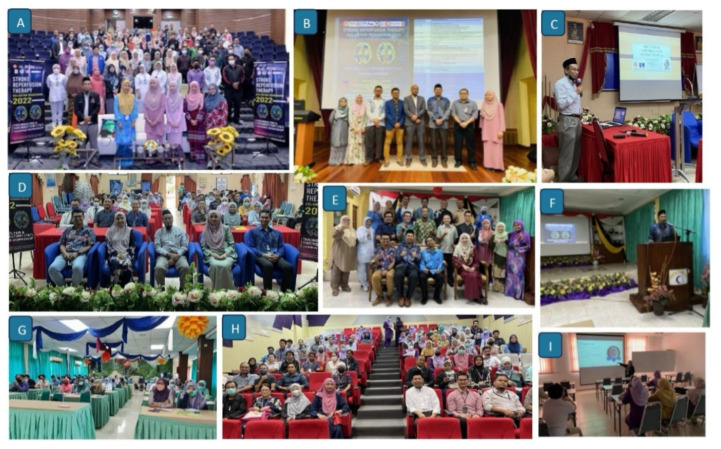
Stroke Reperfusion Therapy Kelantan Roadshow 2022–2023, Thrombolysis and Thrombectomy (T&T) training workshops at the key hospitals in Kelantan: A–B) Hospital Sultan Ismail Petra, Kuala Krai; C–D) Hospital Tanah Merah; E–G) Hospital Tengku Anis, Pasir Putih; H–I) Hospital USM

In collaboration with neurologists from Raja Perempuan Zainab II Hospital, including Dr. Rose Izura Abdul Hamid and Dr. Saidatul Manera Mohd Daud, as well as specialists from district hospitals, the stroke team conducted a series of “Thrombolysis and Thrombectomy (T&T)” training workshops (Figure 9) at key healthcare facilities. These included Hospital Sultan Ismail Petra (Kuala Krai), Hospital Tanah Merah, Hospital Tengku Anis (Pasir Putih), USM Specialist Hospital, and Raja Perempuan Zainab II Hospital. The objective of these workshops was to equip district hospitals with the necessary clinical protocols and operational readiness to function as thrombolysis-capable, stroke-ready hospitals and to strengthen the referral network for thrombectomy-eligible cases to USM Specialist Hospital.

This inter-institutional collaboration led to the establishment of a formalised referral pathway and the delineation of Kelantan into defined “stroke zones,” each linked to a designated stroke referral centre. This framework was developed to streamline acute stroke triage and ensure timely access to reperfusion therapies. As of the current reporting period, four MOH hospitals in Kelantan have achieved stroke-ready hospital status, providing 24/7 intravenous thrombolysis services.

#### Stroke Protocol and Rapid Triage System

**Figure 10:**
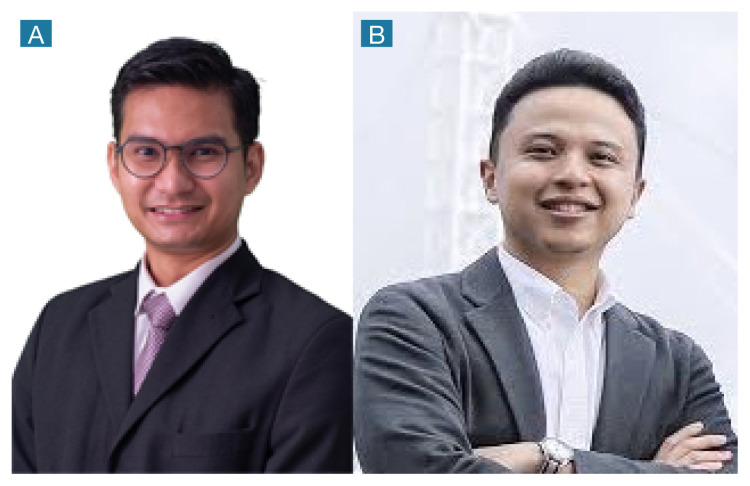
Emergency Physician: A) Dr. Mohamad Masykurin Mafauzy, and B) Dr. Wan Syahmi Wan Mohamad coordinated the stroke pathway in the emergency department

Ischaemic stroke management in the Emergency Department (ED), USM Specialist Hospital, is coordinated by Professor Dr. Kamarul Aryffin Baharuddin, Dr. Mohamad Masykurin Mafauzy, and Dr. Wan Syahmi Wan Mohamad (Figure 10).

**Figure 11:**
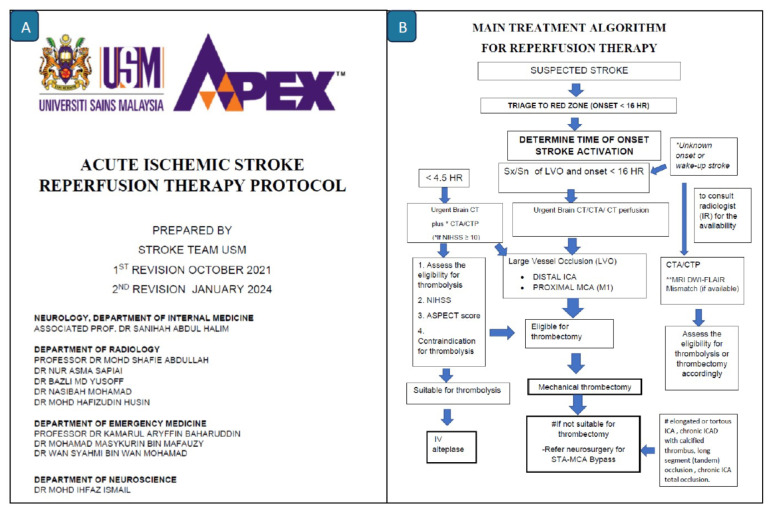
The USM Acute Ischaemic Stroke Reperfusion Therapy Protocol comprises a 16-page set of clinical algorithms and management guidelines, specifically adapted for local implementation

The development and refinement of a standardised stroke protocol and a rapid triage system have been the cornerstones of the enhanced ischaemic stroke service at the ED, USM Specialist Hospital. This protocol was first developed in 2007. The protocol (Figure 11) was revised by the stroke team in 2021 and again in 2024 to streamline care pathways and align with the latest evidence-based guidelines, ensuring prompt diagnosis, prioritisation, and management of suspected acute ischaemic stroke cases (12, 17).

Upon presentation to the ED, patients with a presumptive diagnosis of acute stroke are prioritised and triaged to the “red zone.” A standardised activation protocol, designated as “Stroke Activation,” is implemented for all patients presenting within the therapeutic window for reperfusion therapy. This protocol facilitates the immediate mobilisation of a multidisciplinary stroke team, with activation targeted to occur within 10 min of the patient’s arrival (12, 17).

The initial assessment focuses on accurately establishing the time of symptom onset or the time the patient was last known well, followed by confirming the clinical diagnosis of stroke. For patients arriving within 4.5 h of symptom onset, suitability for intravenous thrombolysis is assessed according to standard clinical guidelines (17). Patients exhibiting clinical signs suggestive of a large vessel occlusion (LVO) undergo further assessment for potential EVT (17).

Hospitalised patients in a non-neurology ward, who develop an acute stroke within the established therapeutic time window, are promptly assessed by the neurology team. Patients who are deemed eligible for reperfusion therapy are either transferred to the ED for intravenous thrombolysis or directly referred for EVT as appropriate.

External hospital referrals are managed using a standardised and expedited Stroke Activation protocol. When logistically feasible, eligible patients are transferred to the USM Specialist Hospital within 30 minutes and go through the same diagnostic and therapeutic workflow as internal cases.

For patients requiring EVT under general anaesthesia with ventilatory support, prior coordination with anaesthesia and critical care teams is initiated to ensure immediate availability.

Post-procedural care is provided in the acute stroke unit (ASU), high-dependency unit (HDU), or intensive care unit for continued monitoring and management, depending on the patient’s clinical condition and severity of neurological impairment.

#### Advanced Neuroimaging Services

**Figure 12:**
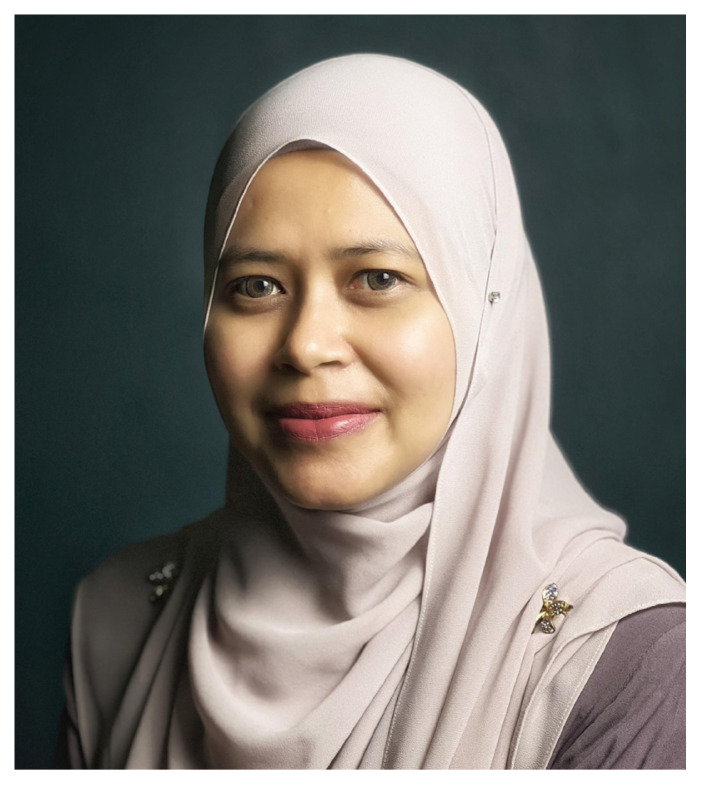
Dr. Nur Asma Sapiai (neuroradiologist) pioneered the implementation of advanced stroke perfusion imaging at USM Specialist Hospital

Neuroradiology services at USM Specialist Hospital are led by Dr. Nur Asma Sapiai (Figure 12), who has completed advanced neuroradiology training in Ankara, Turkey. She pioneered the implementation of advanced perfusion imaging techniques, particularly computed tomography perfusion (CTP), for assessing acute ischaemic stroke within the institution. In 2025, Dr. Nur Asma received an industry-sponsored grant from Penumbra Inc., in partnership with the MOH hospitals in Kelantan and the Kelantan State Health Department. This grant supported a comprehensive stroke education initiative conducted across major hospitals in Kelantan. The stroke education series aimed to enhance awareness and understanding of CTP imaging and its vital role in guiding reperfusion therapies, such as intravenous thrombolysis and EVT, particularly beyond the conventional therapeutic time window.

**Figure 13:**
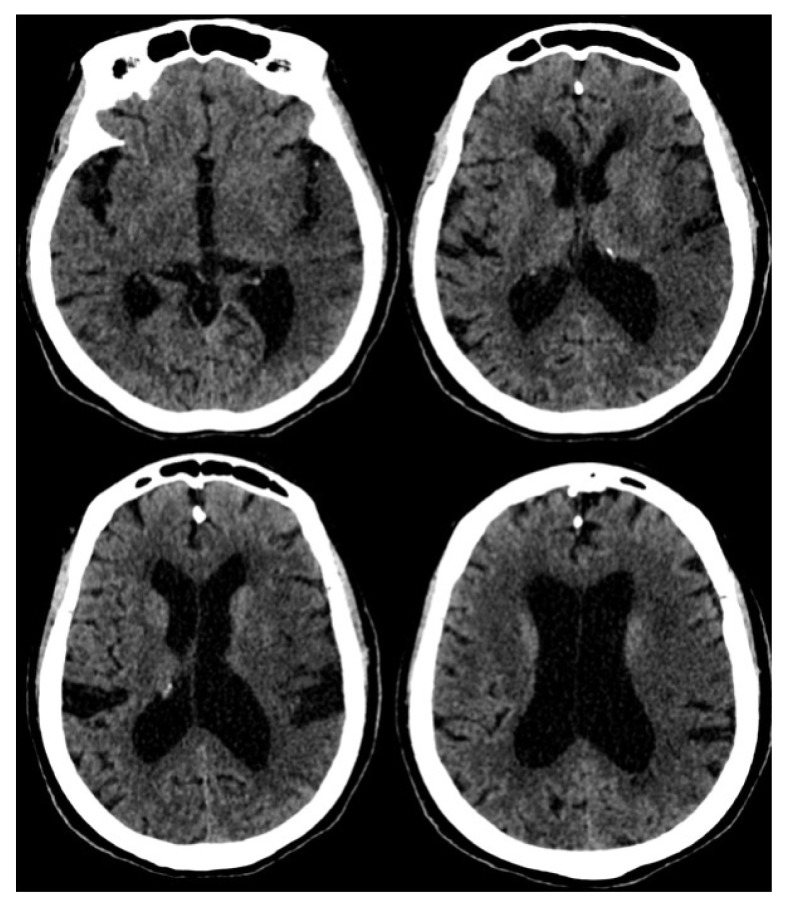
Non-contrast CT brain of Madam M presenting with right hemiparesis. The image shows an ill-defined hypodensity in the left middle cerebral artery territory involving segments M2 to M6 and the left insular cortex. There is loss of grey–white matter differentiation, effacement of sulci, and loss of the insular ribbon sign. No evidence of acute intracranial haemorrhage is seen

Based on the standard protocol, a non-contrast computed tomogram (NCCT) of the brain (Figure 13) is the initial neuroimaging modality in suspected stroke cases; it is performed on an emergent basis, ideally within 20 min of patient arrival (12, 17). NCCT can exclude intracranial haemorrhage and identify early signs of cerebral ischaemia. These signs may include the loss of grey–white matter differentiation, insular ribbon sign, obscuration of the lentiform nucleus, and a hyperdense MCA (18). The Alberta Stroke Program Early CT Score is routinely used to assess early ischaemic changes in the MCA territory, aiding in prognostication and therapeutic decision-making (19).

**Figure 14:**
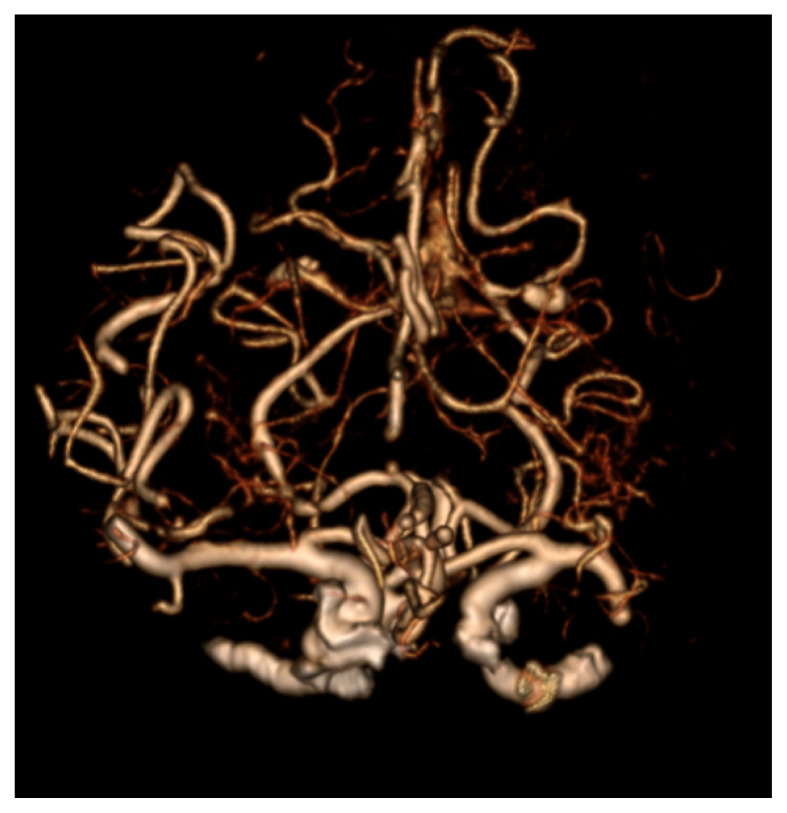
Maximum Intensity Projection image from CT Angiography (CTA) of Madam M demonstrating abrupt tapering at the distal segment of the left M1 branch of the middle cerebral artery, suggestive of a large vessel occlusion

Patients with suspected LVO and absence of intracranial haemorrhage on NCCT immediately undergo CT angiography (CTA) (Figure 14) of the intracranial and extracranial vasculature to assess vascular patency and guide endovascular treatment decisions (20).

CTA is critical for LVO detection, which is a key determinant of eligibility for EVT. CTA also provides essential information on extracranial vasculature, such as carotid artery stenosis and patency of branches, such as the superficial temporal artery (20).

**Figure 15:**
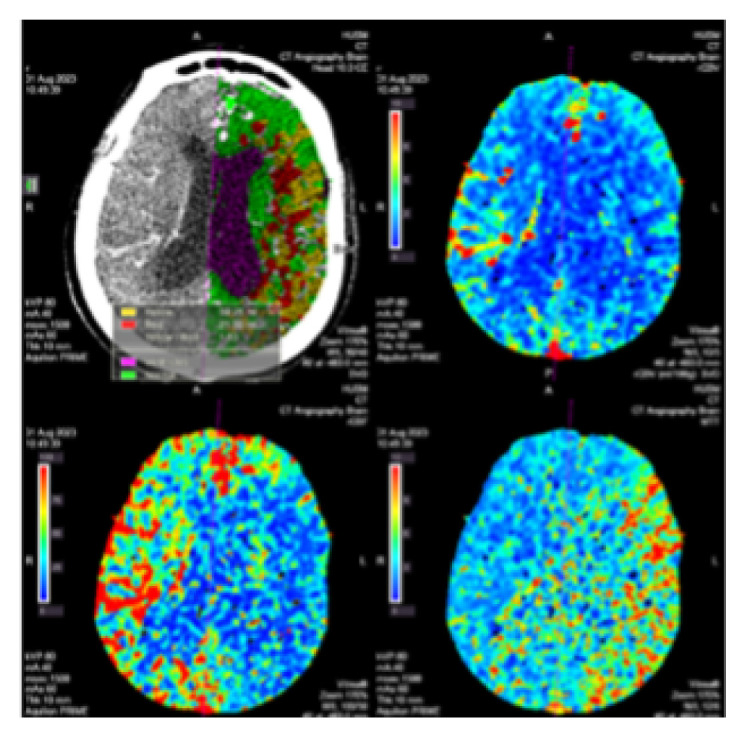
CT Perfusion image of Madam M demonstrating a core infarct surrounded by penumbra in the left M4, M5, and M6 territories. The infarct core volume measures 21.50 mL, with a surrounding penumbra volume of 39.25 mL, yielding a penumbra-to-core ratio of 1.8

CTP imaging (Figure 15) is employed in certain patients, particularly those who are presenting beyond the conventional therapeutic window or have wake-up strokes. CTP estimates and facilitates differentiation between irreversibly infarcted tissue (infarct core) and potentially salvageable ischaemic tissue (penumbra). This individualised, tissue-based approach to reperfusion therapy extends treatment eligibility beyond traditional time thresholds (21).

MRI of the brain is indicated in cases with equivocal CT findings, suspicion of posterior circulation strokes, or a need for enhanced anatomical resolution. Key MRI sequences include diffusion-weighted imaging (DWI) and fluid-attenuated inversion recovery (FLAIR), with the DWI–FLAIR mismatch as a surrogate marker of hyperacute infarction (20, 22). Susceptibility-weighted imaging is conducted to detect cerebral microbleeds and haemorrhagic transformation. MR angiography (MRA) provides a noninvasive assessment of intracranial and extracranial vessels.

Digital subtraction angiography (DSA) is considered the gold standard for cerebrovascular imaging in cases requiring interventional therapy. It offers high-resolution vascular data and enables endovascular procedures, such as mechanical thrombectomy, in real-time (22).

The USM Specialist Hospital employs a standardised, protocol-driven imaging pathway to optimise diagnostic efficiency and clinical outcomes. This algorithm generally begins with NCCT, followed by CTA and, when indicated, CTP. MRI and DSA are performed as needed. A comprehensive 24/7 neuroimaging service supports this workflow, fostering dedicated collaboration among emergency physicians, radiologists, neurologists, and interventional neuroradiologists to ensure the delivery of prompt and effective stroke care.

All imaging data are archived in a centralised Picture Archiving and Communication System, enabling real-time, seamless access by the multidisciplinary stroke team.

#### Intravenous Thrombolysis Services

The emergency medicine and neurology teams co-manage the intravenous thrombolysis service for acute ischaemic stroke. The number of thrombolysis cases (Table 1) has increased in recent years, reflecting improved stroke recognition, streamlined care pathways, and expanded access to advanced neuroimaging modalities (23).

The availability of CTP imaging enables visualisation of the ischaemic penumbra and infarct core; thus, the therapeutic time window for alteplase administration can be extended beyond the conventional 4.5-h limit, up to 9 h from symptom onset in selected patients (17).

At the USM Specialist Hospital, alteplase remains the first-line thrombolytic agent. This drug is provided at the ED. Its standard dosing regimen is 0.9 mg/kg (maximum 90 mg), with 10% administered as an intravenous bolus over 1 min, followed by the remaining 90% infused over 60 min (12, 17). The institutional protocol targets a door-to-needle time of ≤ 60 min to optimise clinical outcomes (12, 17).

The eligibility criteria for intravenous thrombolysis are as follows: age ≥ 18 years, presence of a significant neurological deficit, with National Institutes of Health Stroke Scale score of 4 to 25 points (or lower if symptoms are functionally disabling), neuroimaging evidence excluding intracranial haemorrhage or other contraindications, and absence of high-risk bleeding conditions (12, 17).

Patients receiving intravenous thrombolysis are admitted to the ASU for close observation and continuously monitored for neurological status, blood pressure control, and surveillance for haemorrhagic transformation or other complications for at least 24 h (12, 17). In our centre, follow-up neuroimaging is typically performed at 6 h and after 24 h post-thrombolysis to assess treatment response and exclude early and delayed haemorrhage.

#### Endovascular Thrombectomy Services

**Figure 16:**
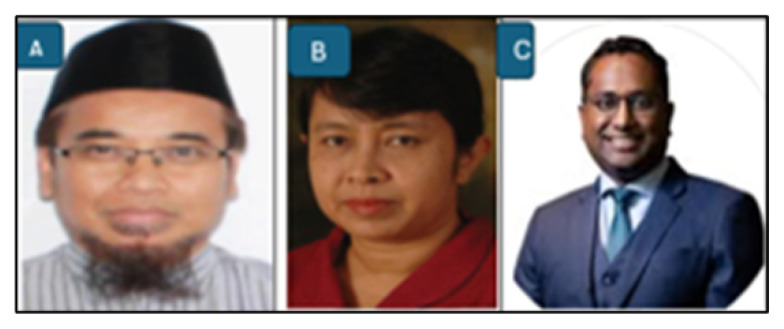
Former interventional radiologist: A) Professor Dr. Mohd Shafie Abdullah and C) Dr. Chandran Nadarajan; and neuro radiologist: B) Dr. Win Mar @ Salmah

**Figure 17:**
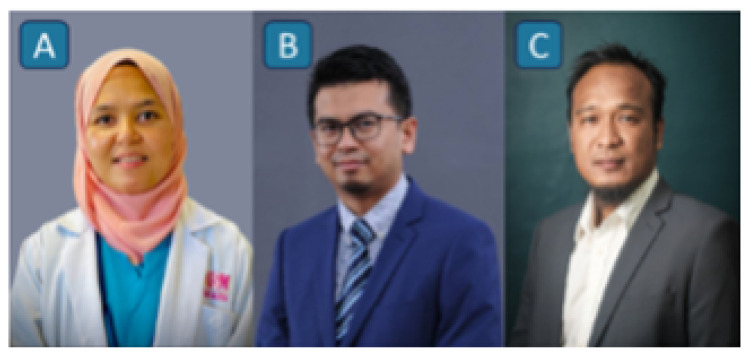
Current interventional radiology team: A) Dr. Nasibah Mohamad (Head); B) Dr. Bazli Md Yusoff; and C) Dr. Mohd Hafizuddin Husin

Professor Dr. Mohd Shafie Abdullah formally established EVT services at USM Specialist Hospital in June 2021 (Figure 16). Currently, three trained interventional operators, namely, Dr. Nasibah Mohamad, Dr. Bazli Md Yusoff, and Dr. Mohd Hafizuddin Husin, can perform thrombectomy procedures (Figure 17).

**Figure 18:**
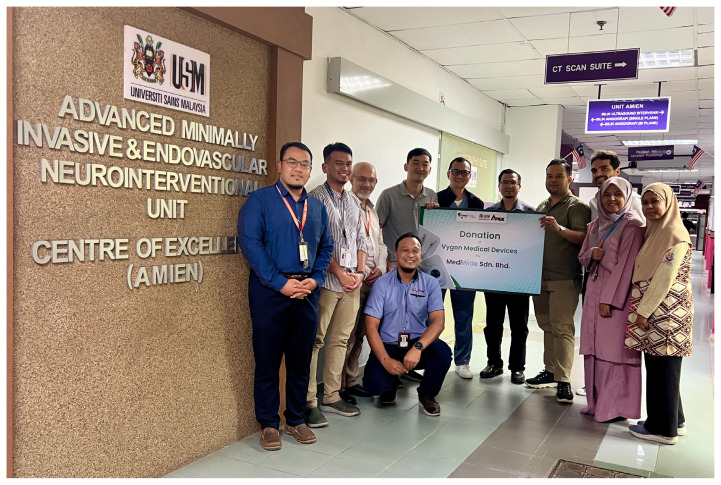
Advanced Minimally Invasive Endovascular and Neuroscience Unit, a specialised facility dedicated to cutting-edge endovascular procedures and advanced neuro-intervention

All EVT procedures are conducted in the department of radiology, using either a single-plane fluoroscopy system or a biplane angiography system located in the Advanced Minimally Invasive Endovascular and Neuroscience Unit (Figure 18), under the leadership of Dr. Nasibah Mohamad.

The interventional radiology unit offers both direct aspiration techniques and stent retriever-based EVT, in alignment with contemporary standards of care (12, 24). EVT candidates must meet the following criteria:

- Presence of clinical signs suggestive of LVO.
- Baseline functional independence, defined as a modified Rankin scale (mRS) score of ≤ 1 before stroke onset.
- Radiological confirmation of LVO with evidence of a favourable perfusion mismatch (i.e., substantial salvageable penumbra with a limited infarct core), as demonstrated by CTA and/or CTP imaging.

**Figure 19:**
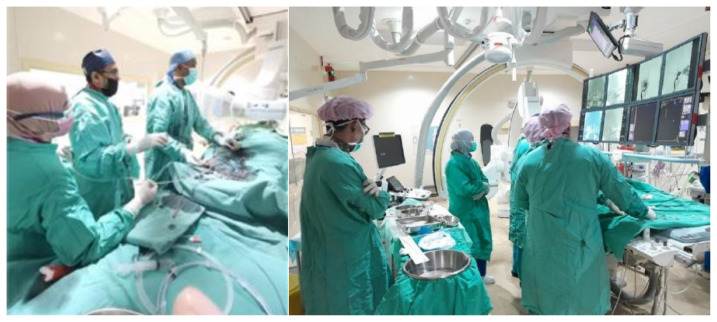
Endovascular thrombectomy procedures performed by interventional radiologists

Once eligibility is confirmed, informed consent is obtained, and preprocedural laboratory and imaging evaluations are rapidly completed. The EVT procedure (Figure 19) is typically performed using aspiration catheters and/or stent retrievers, depending on the anatomical and technical considerations (12, 24).

Following the intervention, patients undergo a repeat neuroimaging to evaluate complications and reperfusion status. Hemodynamic parameters, particularly blood pressure, are closely monitored, and the femoral vascular access site is routinely assessed for signs of hematoma or other complications (12, 17, 24). Subsequently, patients are admitted to the ASU for ongoing neurological monitoring and multidisciplinary care.

**Figure 20:**
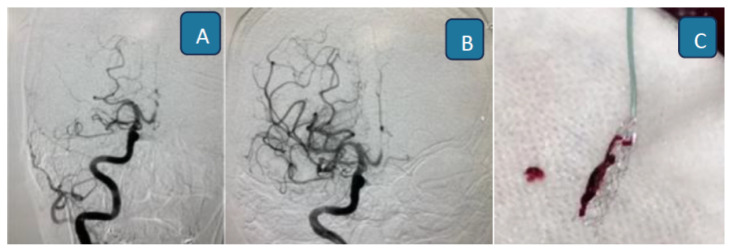
Angiography images show successful reperfusion of the middle cerebral artery: A) Pre-procedure imaging showing vessel occlusion; B) Post-procedure imaging demonstrates restored blood flow; and C) Retrieved blood clot aspirated during the thrombectomy

To date, 40 patients have undergone EVT at USM Specialist Hospital, with 60% achieving successful reperfusion (Figure 20), defined as a modified Thrombolysis in Cerebral Infarction (mTICI) score of 2b or 3. Notably, 17 (42.5%) patients presented within the extended therapeutic time window, supported by advanced imaging selection criteria.

#### ASU Services

The ASU represents a critical component of the multidisciplinary management of acute ischaemic stroke, providing specialised, protocol-driven care designed to optimise neurological recovery and reduce stroke-related morbidity and mortality (12, 17, 25). Evidence consistently demonstrates that admission to a dedicated stroke unit improves functional outcomes, reduces complications, and promotes adherence to guideline-based management (12, 26, 27).

**Figure 21:**
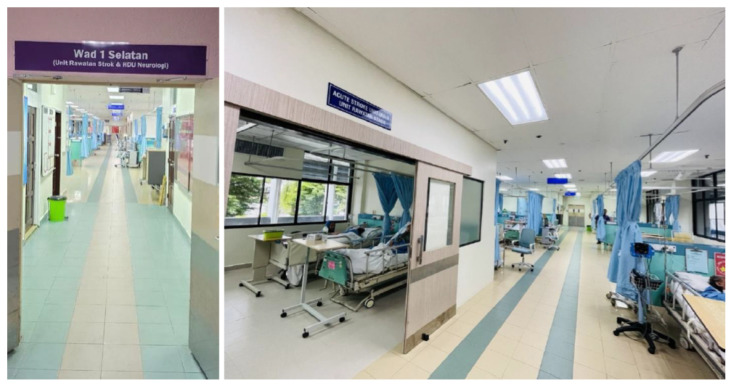
The 25-bedded Acute Stroke Unit at USM Specialist Hospital is dedicated to specialised care and monitoring of stroke patients

At USM Specialist Hospital, the ASU (Figure 21) was established by the neurology unit, led by Associate Professor Dr. Sanihah Abdul Halim, with institutional support from the hospital administration. The unit was formally operational in May 2023. The initial setup consisted of 12 dedicated stroke beds distributed across two designated cubicles within the general medical ward.

**Figure 22:**
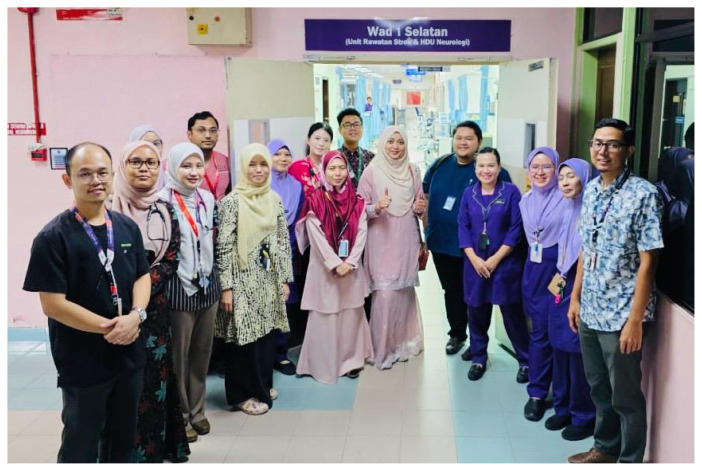
Neurology team, including doctors and nurses, at the Acute Stroke Unit

In November 2024, the unit underwent a significant renovation and structural upgrade to enhance patient care capacity and service delivery. The current configuration includes a HDU with seven beds for patients requiring close hemodynamic and neurological monitoring, a 14-bed dedicated ASU for the management of patients with stable acute stroke, and an additional four rehabilitation beds to support early mobilisation and functional recovery. The unit functions within a multidisciplinary care model comprising neurologists, nursing staff (Figure 22), physiotherapists, occupational therapists, speech therapists, and rehabilitation medicine specialists. This integrated framework facilitates the provision of comprehensive, patient-centred care across the acute and early subacute phases of stroke management.

**Figure 23:**
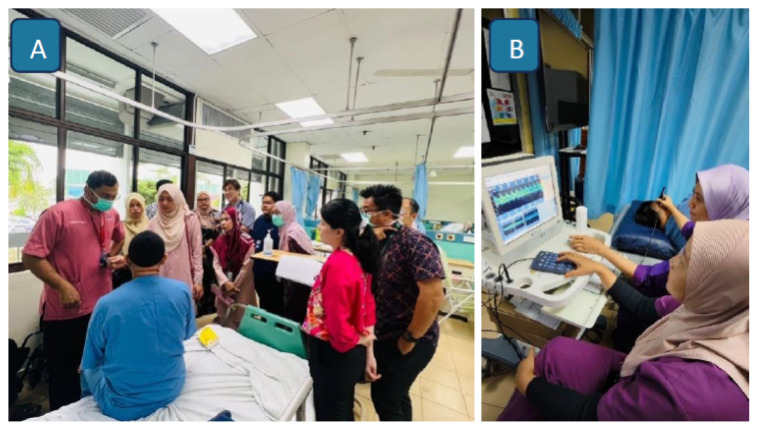
A) Stroke grand rounds are held twice weekly at the Acute Stroke Unit; B) Transcranial doppler ultrasound is used for screening intracranial vascular stenosis

All patients with stroke are admitted to the ASU for a standard duration of 3 to 5 days, depending on clinical stability and rehabilitation needs. During this period, inpatient management (Figure 23A) is directed toward the optimisation of cerebral perfusion and secondary prevention, in line with current evidence-based guidelines (12, 17).

Key management aspects include individualised blood pressure monitoring and intervention and optimisation of intravascular volume status to support adequate cerebral blood flow (12, 17, 28). Glycaemic control and metabolic homeostasis are maintained to minimise secondary neuronal injuries. Measures are also undertaken to prevent complications, such as aspiration pneumonia, urinary tract infections, venous thromboembolism, and pressure injuries. Antiplatelet or anticoagulant therapy is initiated based on stroke subtype and patient risk stratification (12, 17).

A comprehensive aetiological work-up is performed to determine the underlying cause of stroke (12). This includes cardiac evaluations such as electrocardiography, transthoracic or transesophageal echocardiography, and a 24-h or 72-h Holter monitoring to identify arrhythmias or cardioembolic sources. Vascular imaging studies, including CTA, MRA, carotid duplex ultrasonography, and transcranial doppler (TCD) ultrasonography (Figure 23B), are employed to assess both extracranial and intracranial vasculature.

Additional stroke risk factors, such as dyslipidaemia, hypertension, diabetes mellitus, and lifestyle factors, are thoroughly evaluated and addressed through pharmacologic interventions and lifestyle modification counselling (17). Early multidisciplinary rehabilitation is initiated during the ASU stay to facilitate functional recovery and reduce long-term disability.

#### Cerebrovascular Neurosurgery Services

##### Integrated cerebrovascular clinic

The Brain and Behaviour Cluster established the integrated cerebrovascular clinic in January 2019. This clinic provides multidisciplinary, patient-centred care for individuals with complex cerebrovascular disorders. The clinic service was temporarily suspended during the COVID-19 pandemic and resumed coordinated services in early 2023.

**Figure 24:**
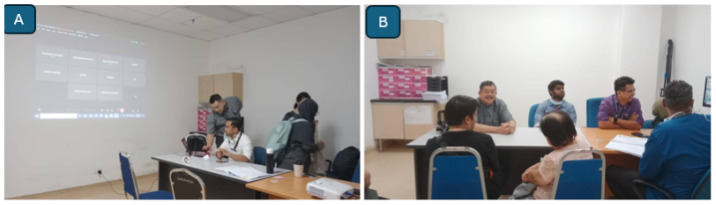
Integrated cerebrovascular clinic: A) Virtual session conducted via Webex; B) Multidisciplinary team session attended by specialists, including Professor Dato’ Jafri Malin (neurosurgeon) and Dr. Bazli (interventional radiology trainee), collaboratively discussing complex AVM case management with the patient’s family member

This integrated clinic facilitates collaborative management by uniting neurologists, interventional neuroradiologists, stereotactic radiosurgeons, and cerebrovascular neurosurgeons. Multidisciplinary sessions are held monthly and involve structured case discussions, enabling comprehensive care planning across healthcare institutions in Malaysia (Figure 24). Meetings are also conducted virtually via Webex, enabling broad participation from regional centres, including Sabah, Perak, Johor, Wilayah Persekutuan, and Selangor.

##### Superficial Temporal Artery to Middle Cerebral Artery (STA–MCA) bypass surgery

**Figure 25:**
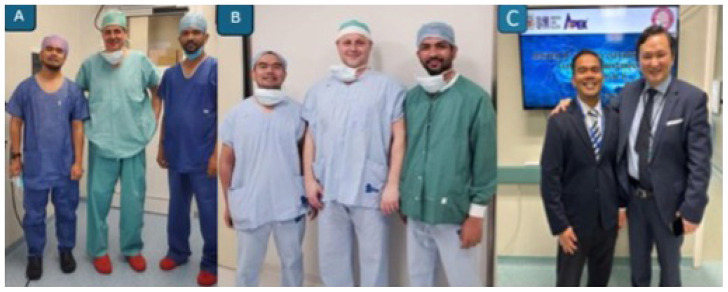
A) Cerebrovascular fellowship training in External Carotid to Internal Carotid (EC–IC) bypass under the supervision of Professor Martin Sames at Masaryk Hospital, Czech Republic; B) EC–IC bypass fellowship under the supervision of Dr. Jiri Fiedler at Ceske Budejovice Hospital, Czech Republic, 2023; C) Professor Bin Xu, a world-renowned cerebrovascular surgeon from Fudan University, Shanghai, China, mentored Dr. Muhammad Ihfaz in bypass surgery

In May 2023, USM Specialist Hospital introduced a pivotal advancement in cerebrovascular surgical practice, following the return of Dr. Muhammad Ihfaz Ismail from his cerebrovascular fellowship training in the Czech Republic (Figures 25A and 25B). During his fellowship, Dr. Ihfaz was mentored in cerebral bypass techniques by renowned cerebrovascular surgeon, Professor Bin Xu (Figure 25C).

**Figure 26:**
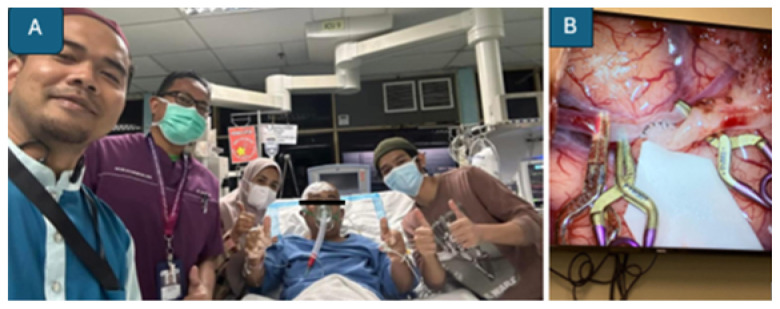
A) First elective patient undergoing STA–MCA bypass in May 2023 for recurrent transient ischaemic attacks caused by chronic right internal carotid artery occlusion. The patient was extubated uneventfully postoperatively with no worsening of neurological deficits; B) Intraoperative image showing the STA–MCA artery anastomosis

**Figure 27:**
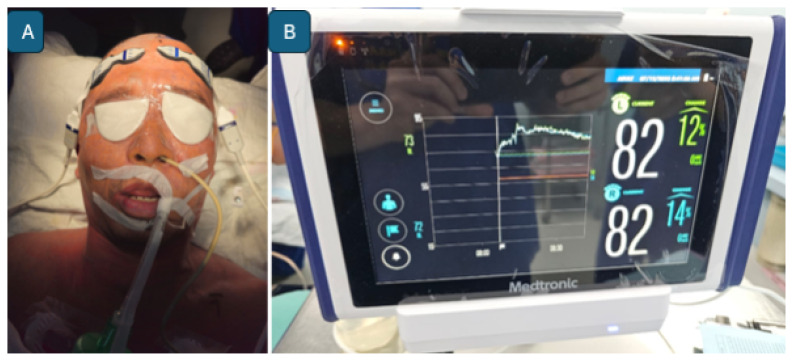
Scalp oximetry monitoring: A) Scalp oximeter applied to both foreheads to measure baseline oxygen concentration levels, indirectly assessing cerebral blood flow to both hemispheres; B) Monitoring device displaying baseline oxygen readings for the right and left brain hemispheres

**Figure 28:**
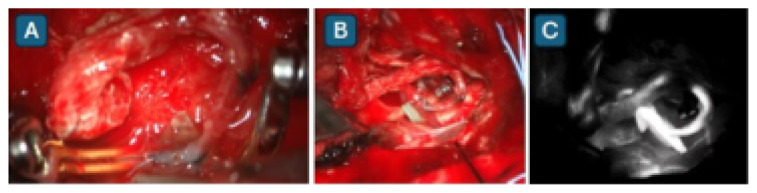
A) Microsurgical anastomosis of the right occipital artery to the tonsillomedullary segment (P3) of the right posterior inferior cerebellar artery (PICA); B) Rescue OA–PICA bypass followed by surgical trapping of a fusiform aneurysm of the right vertebral artery; C) Intraoperative indocyanine green angiography confirming bypass patency

Subsequently, the Department of Neurosciences implemented its first flow-augmentative revascularisation procedure (Figure 26), specifically the STA–MCA bypass with scalp oximetry monitoring (Figure 27). This technique has since been integrated as a third-tier emergency intervention for patients with acute ischaemic stroke presenting with acute LVO, where EVT is either not feasible, such as in acute-on-chronic complete internal carotid artery (ICA) occlusion, or financially inaccessible, or in cases where EVT or intravenous thrombolysis was unsuccessful (29, 30). At our centre, the vascular bypass surgery has also been performed for other indications, such as skull base surgeries necessitating sacrifice of the ipsilateral ICA, and complex aneurysms involving both the anterior and posterior circulations, particularly those requiring an occipital artery to posterior inferior cerebellar artery (OA–PICA) bypass. On 7 August 2025, the neurosurgery team successfully performed the institution’s first rescue OA–PICA bypass, followed by surgical trapping of a fusiform aneurysm of the right vertebral artery (Figure 28). Beyond acute indications, the STA–MCA bypass is also offered for chronic ischaemic cerebrovascular conditions, including intracranial atherosclerotic disease and steno-occlusive disorders such as Moyamoya disease (31, 32).

Notably, before an institutional protocol was established at USM Specialist Hospital, the first STA–MCA bypass surgery at the centre was performed on 12 December 2021, by Dr. Tinesh Kumaran a/l Jayaraman, who was invited from Hospital Kuala Lumpur. The procedure was conducted in collaboration with Professor Dato’ Dr. Jafri Malin Abdullah to treat a patient with Moyamoya disease.

**Figure 29:**
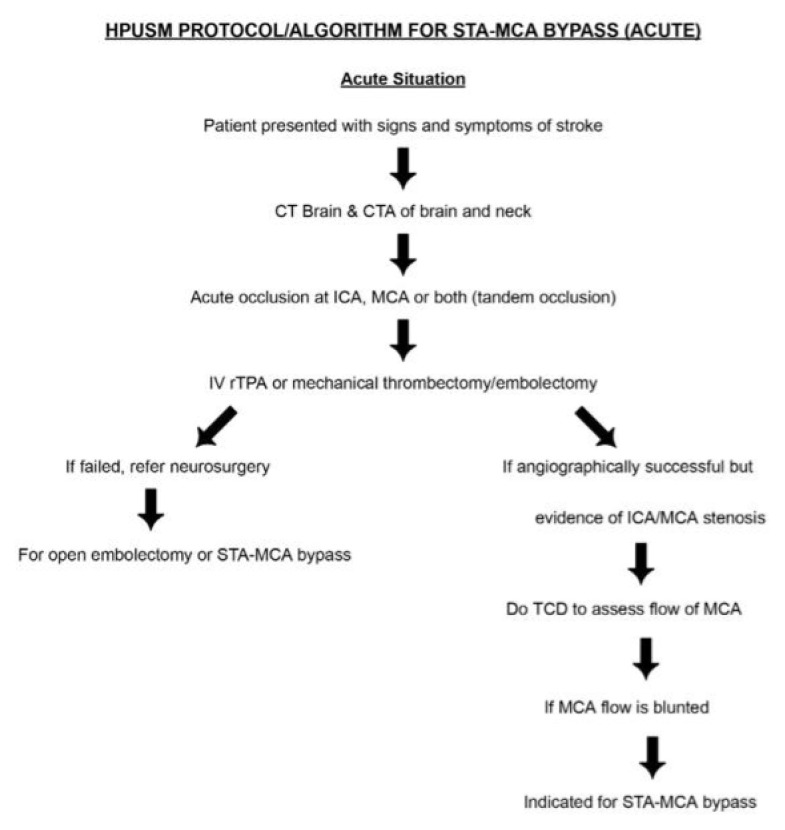
STA–MCA bypass protocol for acute occlusion (Ihfaz Ismail, Zurich Protocol, Masaryk Bypass Protocol)

**Figure 30:**
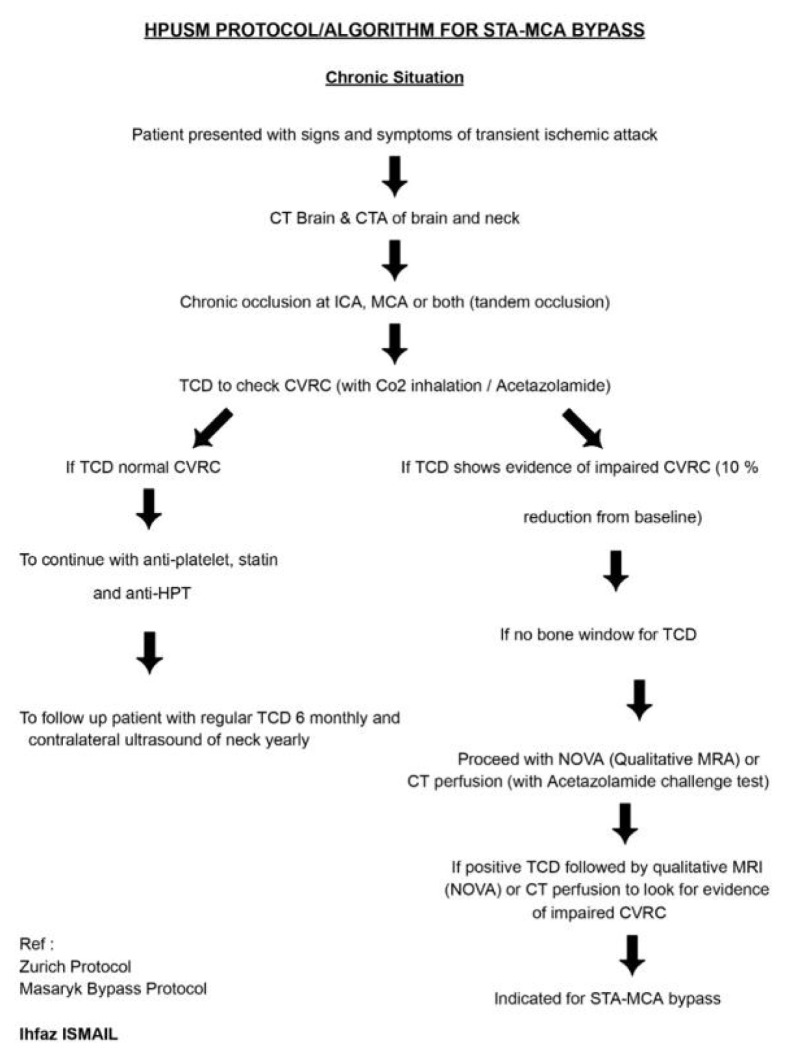
STA–MCA bypass protocol for chronic occlusion

Since 2023, the USM stroke team has formally adopted a new cerebral revascularisation algorithm (Figures 29 and 30), which incorporates STA–MCA bypass as a treatment strategy for patients with acute ischaemic stroke and chronic hemodynamic insufficiency presenting with recurrent transient ischaemic attacks. This approach reflects a paradigm shift toward individualised, physiology-driven surgical intervention in cerebrovascular care.

##### Adjunctive monitoring techniques in cerebrovascular surgery: (TCD) ultrasound with carbon dioxide (CO_2_) reactivity testing and thromboelastography (TEG)

**Figure 31:**
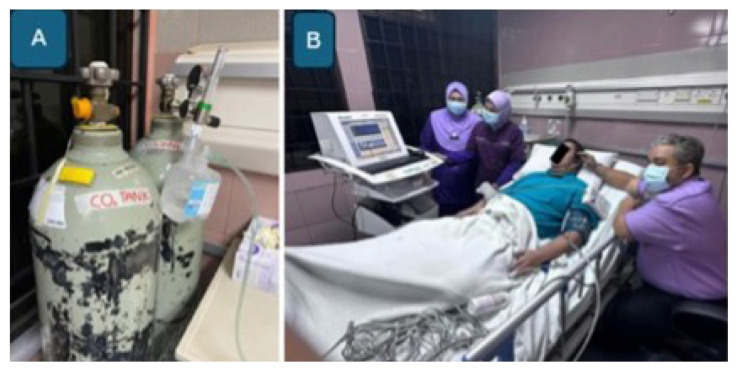
TCD with carbon dioxide provocation test: A) Carbon dioxide (CO_2_) tank; B) Pre and post CO_2_ inhalation measurement of cerebral blood flow. Reduction of blood flow by 10% from baseline indicated that the patient has hemodynamic insufficiency

In addition to extracranial-to-intracranial (EC–IC) bypass, TCD ultrasonography with CO_2_ provocation testing (Figure 31) is conducted as a preliminary screening tool to evaluate cerebrovascular reactivity and confirm hemodynamic insufficiency, which serves as the primary indication for flow augmentation via EC–IC bypass (33).

**Figure 32:**
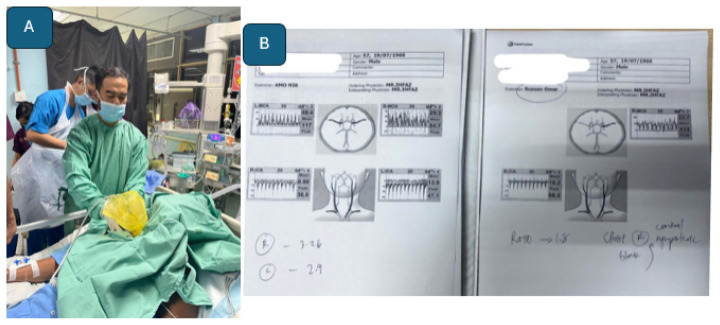
A) Stellate ganglion block performed under sterile technique to treat cerebral vasospasm; B) Transcranial doppler to measure cerebral blood flow pre- and post-stellate ganglion block (immediate response)

Routine TCD ultrasonography is performed to detect cerebral vasospasm early (33). In addition to intravenous nicardipine administration and triple-H therapy (hypertension, hypervolemia, and hemodilution), ultrasound-guided stellate ganglion block (Figure 32) can be administered as an adjunctive intervention to treat cerebral vasospasm.

**Figure 33:**
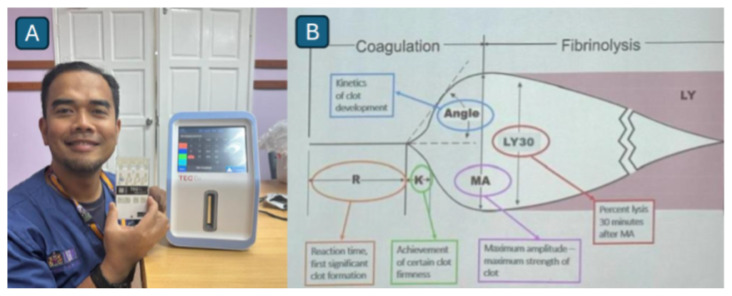
A) The newly acquired thromboelastography (TEG) system offers point-of-care functionality, enabling rapid bedside assessment of coagulation status; B) TEG generates parameters that define the hemostasis process (rate of clot formation, strength, and stability of the clot). It plays a critical role in optimising surgical outcomes by facilitating timely hemostatic interventions and reducing the risk of intraoperative haemorrhage

In addition, the Department of Neurosciences has integrated TEG into clinical practice for nearly two decades, establishing it as a vital component of perioperative management (Figure 33). TEG enables comprehensive assessment of coagulation dynamics, including platelet function, in patients on antiplatelet therapy or those who have recently undergone thrombolysis and require urgent surgical intervention. Additionally, it offers critical insights into qualitative platelet dysfunction and fibrinogen integrity, facilitating the optimisation of hemostasis before surgery.

##### Neurosurgical intervention for cerebral arteriovenous malformation (AVM) and aneurysm

**Figure 34:**
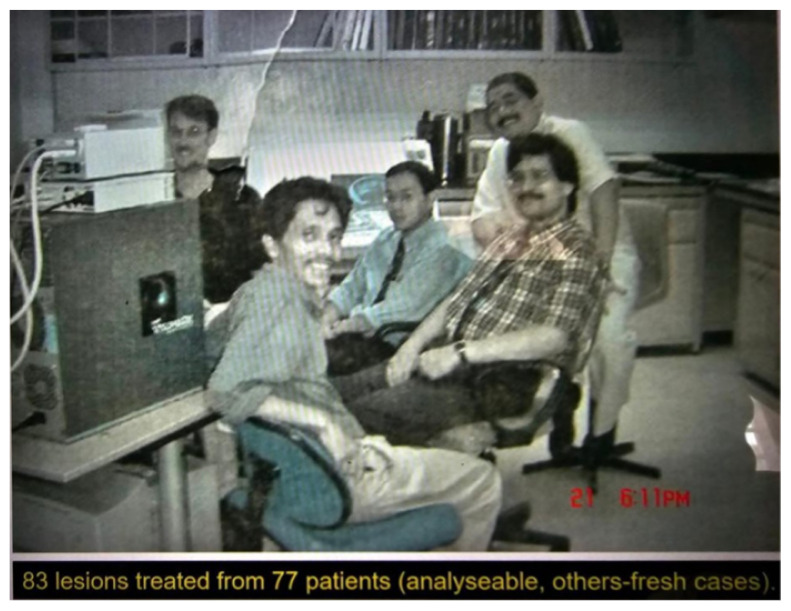
Radiosurgery (SRS), Nik Ruzman Nik Idris (Radiation physicists), Associate Professor Dr. Biwa Mohan Biswal (Oncologist), Professor Zamzuri Idris and Professor Dato’ Dr. Jafri Malin Abdulllah in 2001

Cerebral AVMs and aneurysms represent a significant cause of secondary haemorrhagic stroke. AVM management involves a multimodal approach that includes open microsurgical intervention, radiotherapy, endovascular coiling, and stereotactic radiosurgery (SRS) (34). At USM Specialist Hospital, SRS for AVM was performed in 2001 using the Radionics^®^ Stereotactic Radiosurgery System by Associate Professor Dr. Biswal Mohan and then-junior neurosurgeon Professor Dr. Zamzuri Idris (Figure 34), marking a significant advancement in noninvasive treatment options for complex cerebrovascular malformations. In 2018, Professor Dr. Zamzuri Idris and his team successfully performed the first awake craniotomy for cerebral aneurysm clipping in Southeast Asia, adopting a technique pioneered by internationally renowned neurosurgeon Professor Saleem Abdulrauf (35). This achievement places USM Specialist Hospital among a select group of advanced neurosurgical centres worldwide with the capability to safely conduct this complex procedure. The awake technique facilitates real-time neurological assessment, thereby minimising postoperative neurological complications and reducing the risks associated with general anaesthesia and mechanical ventilation.

#### Neuro-intensive Care Unit

**Figure 35:**
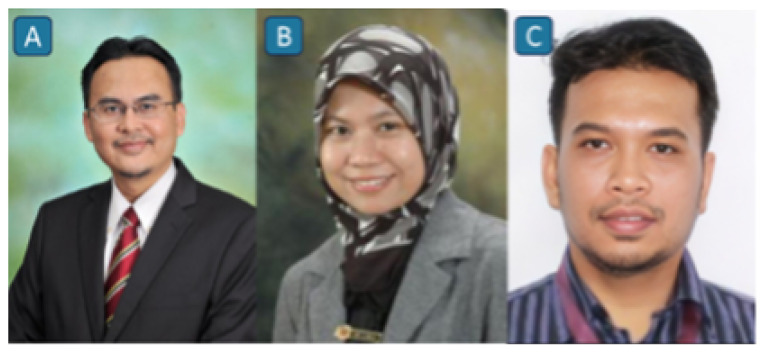
Neuro-intensive care team: A) Associate Professor Dr. W Mohd Nazaruddin W Hassan; B) Dr. Laila Ab Mukmin; C) Associate Professor Dr. Mohamad Hasyizan Hassan

Cerebral hemodynamic monitoring is conducted within a specialised, multimodality neurocritical care unit dedicated to the management of traumatic brain injuries and cerebrovascular emergencies. The unit is supervised by neuro-intensivists (Figure 35), Associate Professor Dr. W Mohd Nazaruddin W Hassan, Dr. Laila Ab Mukmin, and Associate Professor Dr. Mohamad Hasyizan Hassan, under the leadership of the consultant neurosurgeon, Professor Dato’ Dr. Ab Rahman Izaini Ghani.

**Figure 36:**
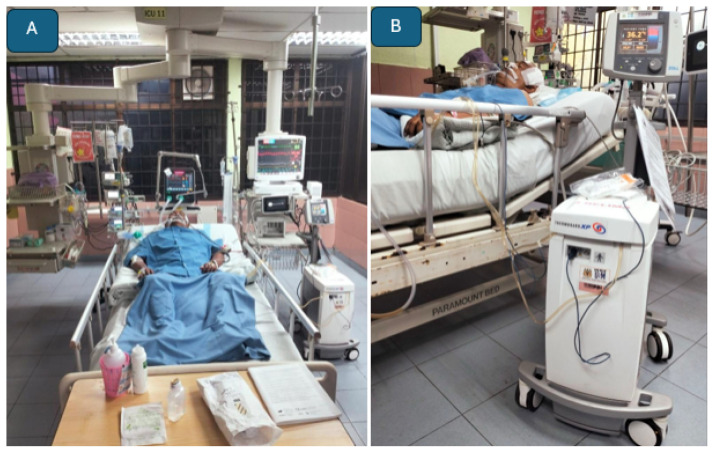
Malignant middle cerebral artery infarction: A) Patient under sedation with continuous intracranial pressure monitoring; B) A systemic cooling machine is used to induce therapeutic hypothermia

The assessment of cerebral hyperemia is supported by advanced neuroimaging modalities, including brain CT, MRI, CTA, MRA, and perfusion studies such as arterial spin labelling (36, 37). These intensive care assessments are typically performed following intraoperative, fluorescence-guided microsurgical interventions for aneurysms and AVMs, as well as intraoperative bypass patency assessments (38). For malignant MCA infarction, the standard clinical practice is to initiate therapeutic hypothermia in conjunction with continuous intracranial pressure monitoring following decompressive craniectomy (Figure 36), as part of comprehensive neuroprotective strategies (39).

#### Comprehensive Neurorehabilitation Services

**Figure 37:**
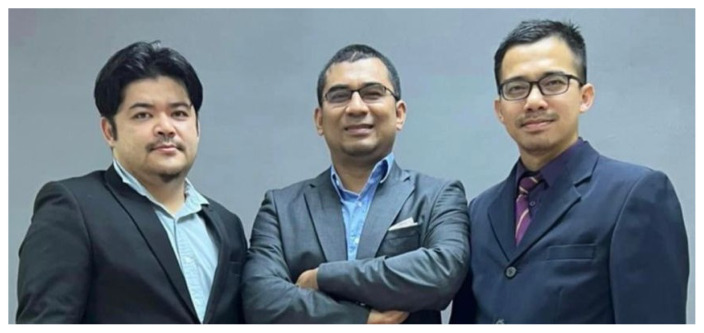
Consultant rehabilitation medicine, from left: Associate Professor Dr. Muhammad Hafiz Hanafi, Dr. Al Hafiz Ibrahim, and Dr. Wan Mohd Aiman Wan Ab Rahman

The neurorehabilitation services at USM Specialist Hospital are led by Associate Professor Dr. Muhammad Hafiz Hanafi, a consultant in rehabilitation medicine, with the assistance of other rehabilitation physicians, namely, Dr. Al Hafiz Ibrahim and Dr. Wan Mohd Aiman Wan Ab Rahman (Figure 37).

**Figure 38:**
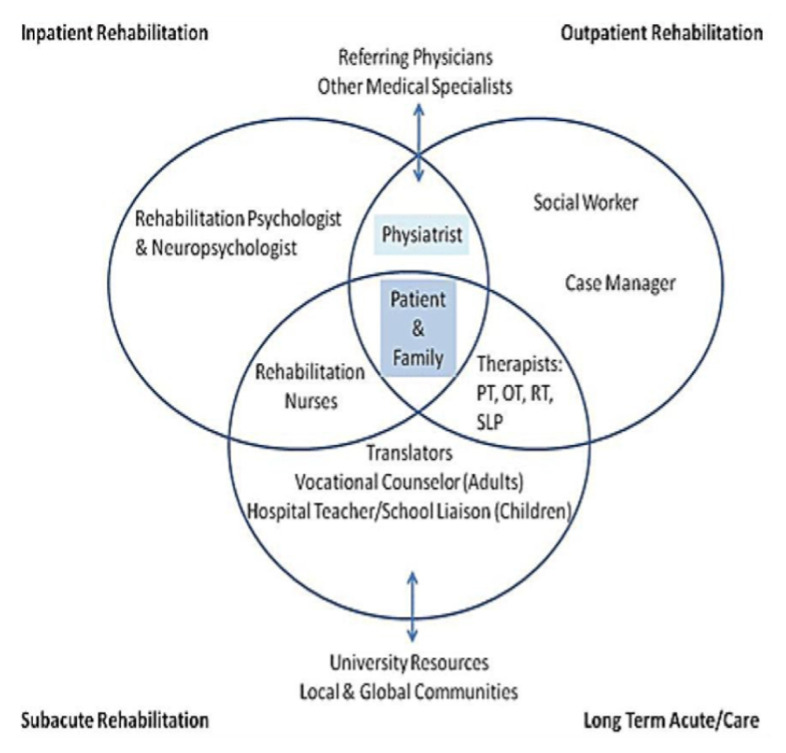
A comprehensive interdisciplinary rehabilitation programme framework at USM Specialist Hospital, integrating multiple specialities for optimised patient recovery

The service is structured as a comprehensive, interdisciplinary programme (Figure 38) that aims to optimise functional recovery and improve the quality of life of patients with neurological impairments, including those recovering from stroke, traumatic brain injury, spinal cord injury, and other neurodegenerative conditions (40).

Patient care is organised within a multidisciplinary framework that includes neurology and neurosurgery consultations, with oversight from rehabilitation medicine for individualised treatment planning. The team integrates physiotherapy, occupational therapy, and rehabilitation nursing to address motor, sensory, and functional deficits. Additionally, specialised services, such as speech and language therapy, neuropsychological evaluation, dietary intervention, and low-vision rehabilitation, are provided to ensure holistic patient care (40).

**Figure 39:**
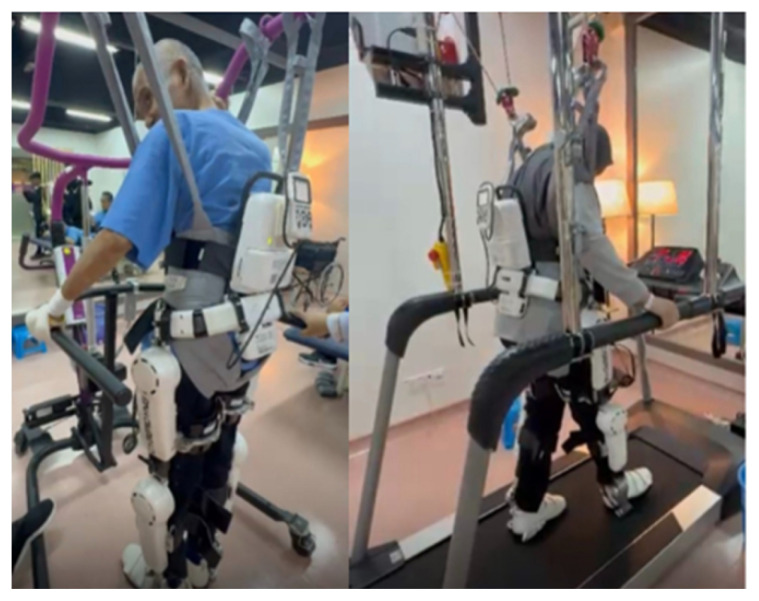
Robotic-assisted rehabilitation designed to support stroke patients with significant disabilities in regaining mobility and function

**Figure 40:**
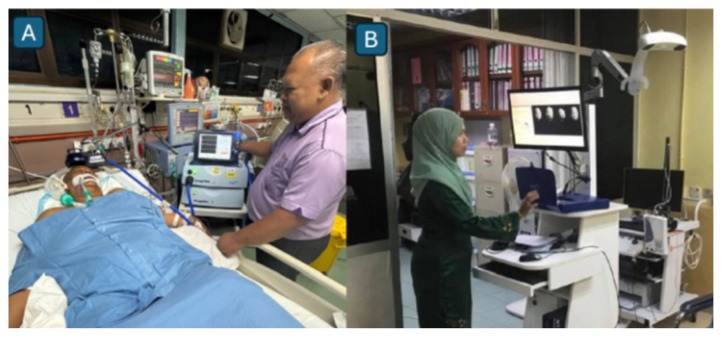
Application of repetitive transcranial magnetic stimulation (rTMS) for post-stroke rehabilitation: A) rTMS session administered in a neurocritical care unit; B) Outpatient rTMS therapy in the maintenance phase aimed at enhancing motor recovery and neuroplasticity in post-stroke survivors

Advanced modalities such as robotic-assisted rehabilitation (Figure 39), neuromodulation therapies such as transcranial magnetic stimulation (Figure 40), and hyperbaric oxygen therapy (HBOT) are incorporated to enhance neuroplasticity and functional outcomes (41, 42). The prosthetics and orthotics unit supports mobility restoration, whereas targeted programmes such as return-to-work, return-to-study, and return-to-drive assist in the reintegration of patients into their daily lives and societal roles.

For patients with central post-stroke pain or hemisensory syndromes, the acute pain service offers tailored pharmacologic and interventional pain management strategies. The team also cooperates with medical social workers to address psychosocial and socioeconomic barriers to recovery, ensuring a continuum of care from acute hospitalisation to community reintegration.

## Stroke Research and Publications

Beyond clinical services, USM has been actively involved in fundamental, translational, and clinical research in the field of stroke for over 25 years. These efforts have been supported by neurosurgical specialists and multidisciplinary teams working in close collaboration across various domains of stroke science (43–100). The hospital’s legacy in comprehensive stroke management and research dates back to the late 1980s, with early contributions from neurosurgeons who laid the foundation for institutional progress. Their achievements until the early 2000s have been documented in prior publications (5, 6)

**Figure 41:**
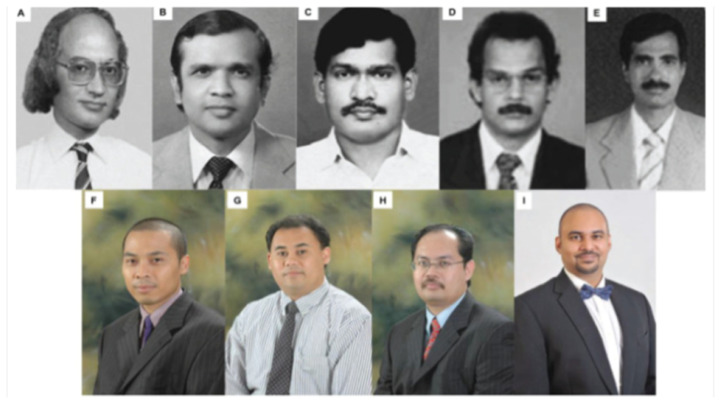
Photos of previous neurosurgeons at USM Specialist Hospital who have contributed significantly to research and publications in the field: A) Dr. Fauzi Ahmad Ali Salem (Egypt; 1984–1987); B) Dr. Benedict Marius Selladurai (Sri Lanka; 1990–1993); C) Dr. Shanmugan Chandrasekaran (India; 1992–1996); D) Dr. Jain George Panattil (India; 2002–2005); E) Dr. Prakash Rao Gollapudi (India; 2004–2005); F) Dr. Sani Sayuthi (2001–2009); G) Dr. Mohamed Saufi Awang (2001–2010); H) Dr. Badrisyah Idris (2004–2019); and I) Dr. Regunath Kandasamy (2012–2020)

Subsequent advancements have been driven by additional key figures in neurosurgery and neurology (Figure 41), including Professor Dr. Zamzuri Idris, Professor Dato’ Dr. Ab Rahman Izaini Ghani, Professor Dato’ Dr. Saufi Awang, Dr. Sani Sayuthi, Dr. Badrisyah Idris, Dr. Regunath Kandasamy, and Professor Dr. John Tharakan, who have continued to advance stroke research at USM (6).

Currently, the institution is engaged in several active research collaborations, including an international trial led by Dr. Muhammad Ihfaz Ismail in partnership with the University of Nottingham, United Kingdom. This study investigates the efficacy of tranexamic acid in patients with intracerebral haemorrhage (the TICH-3 trial).

The stroke team also participates in ischaemic stroke research initiatives and is involved in international multi-centre clinical trials led by Associate Professor Dr Sanihah Abdul Halim, including TRIDENT, OPTIMISTmain, OCEANIC Stroke, and LIBREXIA Stroke, reflecting USM’s ongoing commitment to advancing evidence-based stroke care.

**Figure 42:**
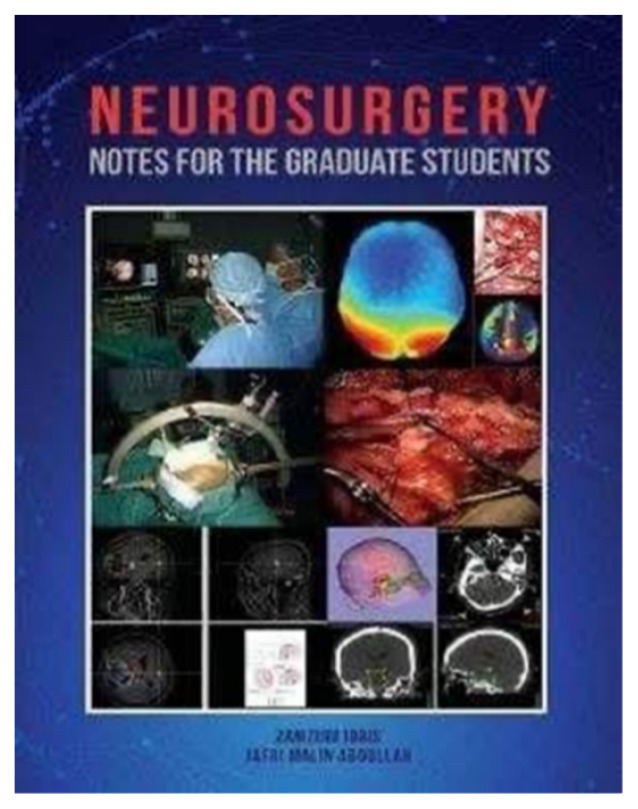
*Neurosurgery* book – notes for the graduate student includes a chapter on stroke neurosurgery

Furthermore, Professor Dr. Zamzuri Idris and Professor Dato’ Dr. Jafri Malin Abdullah have published a neurosurgery book (Figure 42) which contains important notes for graduate students covering all topics, including a chapter dedicated to the surgical management of stroke.

## Stroke Care Challenges at USM Specialist Hospital

Stroke care in low-resource settings is greatly challenged by financial, infrastructural, and systemic limitations, significantly affecting the quality and accessibility of life-saving interventions.

The cost of reperfusion therapies, particularly EVT devices, which are critical for the treatment of LVOs, is one of the most pressing issues. The devices are expensive and not routinely covered by public or private insurance, further restricting access to prompt interventions.

Similarly, the high cost of alteplase, a recombinant tissue plasminogen activator utilised in intravenous thrombolysis, renders this first-line treatment inaccessible to patients without financial resources or insurance coverage, placing the burden on the hospital to source funding through welfare programmes or charitable donations. Consequently, these financial barriers lead to missed therapeutic windows and suboptimal outcomes for certain patients with potentially treatable strokes.

These issues are compounded by the lack of institutional funding for stroke management, which leads to insufficient resources and infrastructure, such as limited access to a 24-h MRI, which is essential for advanced imaging in cases such as wake-up stroke or posterior circulation stroke.

Moreover, there is an insufficiency of trained stroke specialists, nurses, and interventional neuroradiologists. Acute stroke and high-dependency units require upgrades in monitoring systems and equipment. The hospital’s stroke services have yet to participate in any national or international stroke registry systems, which are essential for data collection, epidemiological monitoring, and quality improvement. Additionally, the lack of dedicated space impedes in-house stroke educational and training activities for caregivers and staff, hindering ongoing professional development, caregiver empowerment, and community reintegration efforts.

## Future Directions in Stroke Care at USM Specialist Hospital

As stroke remains one of the leading causes of mortality and long-term disability in Malaysia, USM Specialist Hospital endeavours to advance its stroke services by employing a strategic, multidisciplinary, and evidence-based approach, which includes the following:

- Optimisation of reperfusion therapies by expanding thrombolysis and thrombectomy services with improved access and timeliness.
- Improving the facilities at the ASU and providing a dedicated space for caregiver and staff training.
- Participating in a national or international stroke registry to allow for robust clinical audit, outcome tracking, and quality improvement initiatives.
- Expansion of stroke-related research, encompassing basic, translational, and clinical studies to support innovation and guideline development.
- Strengthening administrative support to increase stroke awareness, prevention, and early intervention.
- Pursuing the international certification as a comprehensive stroke centre, to align clinical practice with global standards, and further enhance the quality of care.

## Conclusion

The evolution of stroke care at USM Specialist Hospital reflects a progressive transformation from basic acute stroke management to the establishment of a multidisciplinary, comprehensive, protocol-driven stroke care model. Despite considerable challenges faced in resource-limited healthcare settings, the hospital has achieved notable progress in stroke service delivery and established a foundation for integrated, high-quality stroke care.

Future priorities focus on optimising reperfusion strategies, improving stroke care facilities, expanding research capacity, participating in stroke registries for outcome benchmarking, and finally pursuing international comprehensive stroke centre certification to position USM Specialist Hospital as a regional centre of excellence in stroke care.

## Figures and Tables

**Table 1:** Number of thrombolysis cases recorded annually from 2012 to 2025, illustrating the trend and growth of acute ischaemic stroke reperfusion therapy over the years

Year	Number of thrombolysis cases
2012	1
2013	3
2014	4
2015	3
2016	2
2017	8
2018	9
2019	14
2020	6
2021	11
2022	15
2023	14
2024	25
2025 (until July)	20
